# Psychological management in chronic headache

**DOI:** 10.1186/1129-2377-14-S1-O11

**Published:** 2013-02-21

**Authors:** Kenneth A Holroyd

**Affiliations:** 1 Ohio University, USA

## 

Behavioral treatments programs for headache and the patient materials (manuals, audiotapes) used in these programs will be described. Various formats for administering behavioral treatments (home-based, phone-based) will be described. Modifications of behavioral management programs for chronic headache and for use in a multidisciplinary team approach rehabilitation of chronic headache will be addressed

